# Two-Tiered Response of Cardiorespiratory-Cerebrovascular Network to Orthostatic Challenge

**DOI:** 10.3389/fphys.2021.622569

**Published:** 2021-03-02

**Authors:** Peter Mukli, Zoltan Nagy, Frigyes Samuel Racz, Istvan Portoro, Andras Hartmann, Orestis Stylianou, Robert Debreczeni, Daniel Bereczki, Andras Eke

**Affiliations:** ^1^Department of Physiology, Semmelweis University, Budapest, Hungary; ^2^Vascular Cognitive Impairment and Neurodegeneration Program, Oklahoma Center for Geroscience and Healthy Brain Aging, Department of Biochemistry and Molecular Biology, University of Oklahoma Health Sciences Center, Oklahoma City, OK, United States; ^3^Institute of Translational Medicine, Semmelweis University, Budapest, Hungary; ^4^Institute for Globally Distributed Open Research and Education (IGDORE), Stockholm, Sweden; ^5^Department of Neurology, Semmelweis University, Budapest, Hungary; ^6^Department of Radiology and Biomedical Imaging, Yale University School of Medicine, New Haven, CT, United States

**Keywords:** network physiology, orthostatic stress, cardiorespiratory, cerebrovascular, near-infrared spectroscopy, transcranial Doppler, nonlinear, surrogate testing

## Abstract

Dynamic interdependencies within and between physiological systems and subsystems are key for homeostatic mechanisms to establish an optimal state of the organism. These interactions mediate regulatory responses elicited by various perturbations, such as the high-pressure baroreflex and cerebral autoregulation, alleviating the impact of orthostatic stress on cerebral hemodynamics and oxygenation. The aim of this study was to evaluate the responsiveness of the cardiorespiratory-cerebrovascular networks by capturing linear and nonlinear interdependencies to postural changes. Ten young healthy adults participated in our study. Non-invasive measurements of arterial blood pressure (from that cardiac cycle durations were derived), breath-to-breath interval, cerebral blood flow velocity (BFV, recorded by transcranial Doppler sonography), and cerebral hemodynamics (HbT, total hemoglobin content monitored by near-infrared spectroscopy) were performed for 30-min in resting state, followed by a 1-min stand-up and a 1-min sit-down period. During preprocessing, noise was filtered and the contribution of arterial blood pressure was regressed from BFV and HbT signals. Cardiorespiratory-cerebrovascular networks were reconstructed by computing pair-wise Pearson-correlation or mutual information between the resampled signals to capture their linear and/or nonlinear interdependencies, respectively. The interdependencies between cardiac, respiratory, and cerebrovascular dynamics showed a marked weakening after standing up persisting throughout the sit-down period, which could mainly be attributed to strikingly attenuated nonlinear coupling. To summarize, we found that postural changes induced topological changes in the cardiorespiratory-cerebrovascular network. The dissolution of nonlinear networks suggests that the complexity of key homeostatic mechanisms maintaining cerebral hemodynamics and oxygenation is indeed sensitive to physiological perturbations such as orthostatic stress.

## Introduction

The original concept of homeostasis (Bernard, 1858) refers to the concerted actions of physiological processes in assuring the constancy of the “*milieu intérieur*,” the internal environment of the organism. Dynamic interdependencies within and between physiological systems and subsystems are essential for the organism to maintain its steady-state via a plethora of homeostatic mechanisms (Cannon, 1929). Due to continuous perturbations, the system operates in non-equilibrium with its controlled parameters drifting away from their set-point, which characterizes an *optimal* or desired state of the system. In contrast to this single steady-state concept (Cannon, 1929), a physiological system in fact exhibits dynamic fluctuations even under natural conditions that can be readily traced across a homeostatic space, which is a multidimensional metric space spanned by a set of regulated parameters (Keramati and Gutkin, 2014). In this state-space representation, the resting-state dynamics of the system—emerging from active (physiological) and passive (physical) processes—evolves along a trajectory embedded in a *homeodynamic* space (Rattan, 2014). Perturbations elicit regulatory responses that attempt to restore the optimum of the internal environment. Such control of physiological parameters is usually achieved by multiple negative feedback loops that feature a distribution of time scales resulting in a delayed response typically with an amplitude proportional to the deviation from the set-point (Ivanov et al., 1998; Ashkenazy et al., 2002; Lo et al., 2002).

An abrupt change in body position triggering a rapid fluctuation in central arterial pressure can be viewed as a typical perturbation, which activates circulatory adaptation mechanisms that are essential to stabilize cerebral perfusion. Indeed, in a wide range of arterial blood pressure, global cerebral blood flow is tightly regulated by the cerebrovascular system; a phenomenon referred to as pressure autoregulation (Lassen et al., 1978). To a large extent, tolerance to the orthostatic stress also invokes autonomic control adjustments by baroreflex mechanisms that accelerate heart rate (Chapleau, 2012). This will stabilize blood pressure and prevent the transient hypoperfusion in the brain that otherwise would result in orthostatic syncope. In addition to its influences on the vasomotor tone at specific frequencies (0.1 Hz, Traube-Hering-Mayer-wave; Julien, 2006) the autonomic nervous system provides coordination between cardiac and respiratory dynamics—such as respiratory sinus arrhythmia and cardiorespiratory phase synchronization (Bartsch and Ivanov, 2014) –, which is essential for optimal performance of these transport systems. In summary, the interactions between and within cardiorespiratory and cerebrovascular systems are established by distinct mechanisms operating at different time scales, thus bringing about coupling of various type and strength. The recently introduced concept of Network Physiology offers a novel framework for an enhanced characterization, quantification, and understanding of the dynamical interactions between organ systems underlying homeostatic adaptation (Bashan et al., 2012; Bartsch et al., 2015; Lin et al., 2020). In this concept, a network is created by organ systems, represented by nodes, each having complex output dynamics; whose functional interactions are captured in measures of interdependencies and are represented by edges between nodes.

According to this novel perspective, a thorough characterization of the investigated physiological systems can be achieved by defining this network as a representation of the actual state in the homeodynamic space. Furthermore, the network response to a specific challenge, such as orthostatis in our case or mental workload (Zanetti et al., 2019), could reveal integrated and quantitative features of dynamic adaptation of the organism to specific perturbation.

In this study, we examined the cardiorespiratory-cerebrovascular network by assigning simple relationship measures as links for quantifying linear and nonlinear interactions. We use this framework to test the hypothesis that postural changes induce alterations in the topology of cardiorespiratory-cerebrovascular network. We report on a frequency-specific linear and nonlinear network response to a sudden change in body position and we address the question whether topological changes in these networks could indicate altered physiological regulation.

## Materials and Methods

### Participants

This study was approved by the Regional and Institutional Committee of Science and Research Ethics of Semmelweis University (ethical approval number: 53/2009) and was conducted in compliance with the Helsinki Declaration. A total of 10 healthy young adults were recruited for participation in this study; none of them reported neurological, psychiatric or cardiovascular diseases or living with the condition of orthostatic hypotension. Five-five female (age: 26.2 ± 4.6 years, height: 1.67 ± 0.07 m weight: 58.8 ± 9.7 kg BMI: 20.9 ± 2.6) and male subjects (age: 26.6 ± 3.8 years, height: 1.79 ± 0.07 m weight: 76.0 ± 9.7 kg BMI: 23.9 ± 2.5) participated in the study. Two female subjects were taking oral contraceptive regularly. One male subject was excluded due to a lack of acoustic window necessary for transcranial Doppler (TCD) measurements (see below). Written informed consent was obtained from all subjects prior to participation.

### Measurement Protocol

Mean arterial blood pressure (MAP) was monitored continuously and non-invasively by an array of transducers according to the tonometric principle (Colin BP-508, Colin Medical Technology Corporation, Komaki City, Japan). The subject’s left wrist was positioned in an elastic brace that was secured firmly but comfortably with straps. The tonometer was placed over the radial bone of the participant in a manner that at least three adjacent sensors detected pulsations from the radial artery by an oscillometric measurement at navel height. Breathing was recorded by an uncalibrated capnograph (Colin BP-508, Colin Medical Technology Corporation, Komaki City, Japan) using a soft plastic mask, which was mounted on the subject’s face. Blood flow velocity (BFV) in the left and right middle cerebral arteries (MCAs) was monitored by TCD sonography. The transducers (2-MHz pulsed-wave DWL Multidop-T, Sipplingen, Germany) were fitted on an elastic headband that was adjusted to obtain signals from 35 to 60 mm depth for a range of linear flow velocities between 50 and 74 cm/s (Aaslid et al., 1982). The above-described analog signals were relayed to a data acquisition device (DT9816, Data Translations, Marlborough, Massachusetts, United States) for sampling with a frequency of 100 Hz (Winview LE, Team Solutions Inc., Grande Vista Ave Laguna Niguel, California, United States).

**FIGURE 1:**
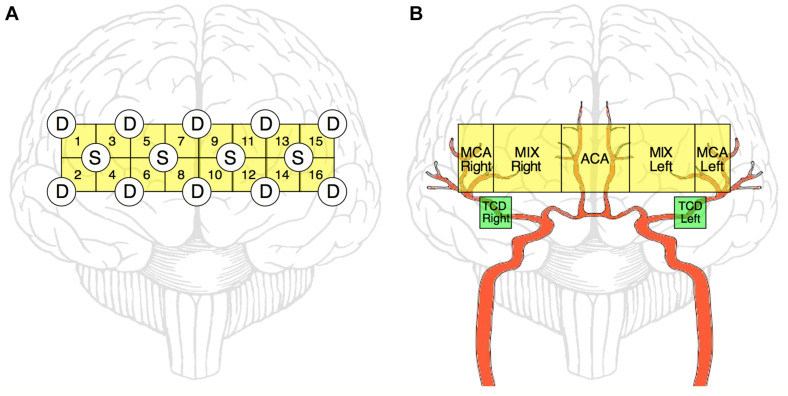
Schematic representation of NIRS and TCD measurements. NIRS-optode layout over the prefrontal cortex **(A)**. Four light-emitting diode sources (S) and ten photodetectors (D) are arranged in a square array forming 16 channels altogether. Of these 16 channels, ultimately 5 regions of interests are defined (by averaging the obtained relationship parameters within that region) to reflect the features of blood supply in the prefrontal cortex (**B**, yellow blocks). The main relevant arterial supply routes are shown in red. MCA Right, right middle cerebral artery; MCA Left, left middle cerebral artery; ACA, anterior cerebral artery; MIX Right, region supplied by MCA Right and ACA; MIX Left, region supplied by MCA Left and ACA; TCD Right and Left, sites of transcranial Doppler sonography measurement (**B**, green blocks).

Cerebrocortical hemodynamic fluctuations were monitored continuously by near-infrared spectroscopy (NIRS) (Bunce et al., 2006). We employed a 16-channel continuous-wave NIRS research instrument (by courtesy of Professor Britton Chance; NIM Inc., University of Pennsylvania, Philadelphia, United States) equipped with a set of four light emitting diodes operating at three different wavelengths (730, 805, and 850 nm) (Chance et al., 2007) and a set of 10 photodiodes with 2.5 cm separation from their corresponding light source (resulting in a 1.25 cm penetration depth, see Figure 1A; Firbank et al., 1998). Before measurement, the optode was mounted on the forehead with appropriate shielding from ambient light and its position was secured with Velcro. Hence, light intensities were measured from 16 regions of the prefrontal cortex (PFC) and were converted into digital signals with a sampling rate of 3 Hz.

The measurements took place in a darkened room with participants seated in a comfortable armchair. The protocol was adopted from the work of Narayanan et al. (2001) and it consisted of 30-min resting awake period with eyes open while seated with feet touching the floor (*resting*). When instructed, the subjects quickly rose to an upright position for 1 min (*stand-up*). Subsequently, the participants were instructed to sit quickly with the measurement continuing for another minute in this position (*sit-down*).

### Data Preprocessing

Data preprocessing and analysis were carried out in MATLAB (The Mathworks, Natick, MA, United States) using custom scripts written by authors and taken from the BP_Annotate toolbox. Cardiac cycle duration time series (CCD, using an estimate of RR-interval) was derived from the blood pressure recording using the algorithm described in Pan and Tompkins (1985) and Sun et al. (2006). From the respiratory record, we created the breath-to-breath interval time series (BB) by using the *peakfinder* function of MATLAB. Finally, TCD, CCD, and BB time series (signals for short) were resampled at 3 Hz and synchronized with the NIRS signals using time stamps corresponding to light intensity values and markers set during the measurement protocol.

Cerebrovascular time series were preprocessed to attenuate the contribution of blood pressure changes that would establish an obvious link when investigating their relationships. Accordingly, we estimated the BFV signal component as a linear derivate of blood pressure oscillations by adopting a spectral analytical approach (Zhang et al., 1998). To calculate the power spectral densities, we used the Welch method with a window width of 128 data points and 50% overlap, and estimated the transfer function between changes in arterial blood pressure and cerebral blood flow velocity from their spectra. Filtered BFV was then determined according to

(1) $${\text{BFV}}_{f\,i\,l\,t\,e\,r\,e\,d}=B\,F\,V-A\,B\,P⊗\left[{ℱ}^{-1}\,\left(S_{A\,B\,P-B\,F\,V}/S_{A\,B\,P}\right)\right]$$

where ⊗ is the convolution operator, ℱ^−1^ denotes the inverse Fourier-transformation, *S*_*ABP–BFV*_ is the power of cross-spectral density of the ABP and BFV signals and *S*_*ABP*_ is the power of auto-spectral density of the ABP signal.

NIRS channels with out of range gain values indicated poor contact quality and thus were excluded from further analyses. These cases of low signal-to-noise records were also confirmed by visual inspection that eventually resulted in 13–16 channels kept for the subsequent analysis. A discrete wavelet filter was applied to remove motion artifacts from the measured light intensity signals for each source-detector pair and each wavelength (Molavi and Dumont, 2012). A fifth-order zero-phase Butterworth filter (Kirilina et al., 2013) was utilized to bandpass filtering in the frequency ranges for representing low- and high-frequency components of the NIRS signal (Tian et al., 2009): 0.02–0.4 Hz (LF), 0.4–1.5 Hz (HF). While HF component mainly reflects contribution from respiratory and cardiac cycle, LF component originates from endothelium-related metabolic activity, neurovascular coupling, vasomotion and autonomic control (Li et al., 2013). Optical density (OD) as a peripheral component was then identified based on the procedure outlined in Mesquita et al. (2010). Taking the ABP signal as a regressor, we estimated its contribution to the NIRS signal and subtracted it from the measured changes (Δ) in optical density, yielding a filtered signal:

(2) $$\text{Δ}\,O\,D_{f\,i\,l\,t\,e\,r\,e\,d,n}=\text{Δ}\,O\,D_{n}-A\,B\,P⊗\left[{\left(A\,B\,P^{T}\cdot A\,B\,P+\lambda^{2}\,I\right)}^{-1}\,A\,B\,P^{T}\cdot \text{Δ}\,O\,D_{n}\right]$$

where Δ*OD*_*n*_ is the observed change in optical density of the *n*-th channel influenced by ABP considered as a regressor; *I* denotes the identity matrix, superscript *T* denotes transpose operation and λ is the regularization parameter that was set to 0.1 times the maximum of the diagonal elements of *ABP^T^*⋅*ABP*. Subsequently, concentration changes of total hemoglobin in the brain tissue were calculated by the revised form of the modified differential Beer-Lambert law (Cope et al., 1988; Kocsis et al., 2006; Cooper et al., 2012), yielding a total tissue hemoglobin concentration time series denoted as HbT. Finally, we further enhanced the component of NIRS signals associated with neurovascular coupling by performing correlation-based signal improvement (CBSI) (Cui et al., 2010) that is an additional procedure aimed at eliminating artifacts unrelated to resident processes of regional hemodynamics, such as motion artifacts.

### Reconstructing Cardiorespiratory-Cerebrovascular Networks

We selected 30 non-overlapping artifact-free segments from resting, one from the stand-up and one from the sit-down period, with duration of 50 s each. The starting points were chosen 5 s after the stand-up/sit-down maneuver in the task periods. Dependencies between signals were assessed between the standardized time series and a population of surrogate time series pairs (*n* = 40) that were generated to preserve all properties of the original pair but the tested one. The presence of linear or nonlinear dynamics was evaluated separately by statistically comparing the relationship measure obtained from the original pair to its distribution derived from the surrogate population. This approach enabled statistical assessment of changes in the reconstructed physiological networks after postural changes also at the individual level.

A network reflecting the strength of *linear* relationships between the concerned physiological signals was reconstructed in each frequency band using cross-correlation analysis (low: 0.02–0.4 Hz, high: 0.4–1.5 Hz). The Pearson-coefficients (*r*) were determined using the entire selected time period (of 50-s length) according to:

(3) $$r=\frac{\sum_{i=1}^{w}\left(X_{i}-\bar{X}\right)\,\left(Y_{i}-\bar{Y}\right)}{\sqrt{\sum_{i=1}^{w}{\left(X_{i}-\bar{X}\right)}^{2}}\,\sqrt{\sum_{i=1}^{w}{\left(Y_{i}-\bar{Y}\right)}^{2}}},$$

where *X* and *Y* represent the physiological processes of interest and *r* follows a Student’s t-distribution. Each pair of time series were compared with pairs from uncorrelated bivariate normal distribution with the aid of *t*-test, yielding a *p*-value for each *r*; only signal pairs with significant (*p* < 0.05) correlation was used in the calculation of network.

Another network consisting of the same physiological signals was reconstructed in each frequency band using cross mutual information analysis (Shannon, 1948; Steuer et al., 2002), which can capture both linear and nonlinear dependencies. Having two time series (*X* and *Y*) of length *N*, we first replaced their numerical values by their rank order—thus converting *X* and *Y* to *A* and *B*, respectively –, and plotted the obtained ranks in perpendicular axes. Each axis was partitioned into *z* smaller components called elements, with *A*_*i*_ and *B*_*j*_ representing the *i*-th and *j*-th element of the *X* and *Y*, respectively. Each element contained *N*_1_ data points except the *z*-th element, which contained *N*_2_ datapoints. The intersection of the *A*_*i*_ and *B*_*j*_ element creates the (*A*_*i*_, *B*_*i*_) grid. The partitioning was carried to yield 5 data points in each grid, except the grids of the last elements of each axis that contained 5 or fewer data points. Then *C*_*ij*_ was obtained as the sum of every value of the data points in (*A*_*i*_, *B*_*i*_). Finally, the mutual information (MI) between the two time series was obtained by:

(4) $$M\,I\,\left(X,Y\right)=\sum_{i}^{z}\sum_{j}^{z}\frac{C_{i\,j}}{N}\,l\,o\,g_{2}\,\left[\frac{C_{i\,j}\,N}{N\,\left(i,j\right)}\right],$$

where *N*(*i*,*j*) = *N*_1_*N*_2_ if *i* = *z* or *j* = *z*, $N\,\left(i,j\right)=N_{2}^{2}$ if *i* = *j* = *z* and $N\,\left(i,j\right)=N_{1}^{2}$ in any other case. The obtained MI value was then assigned to each recorded pair of physiological processes using the same selected segments as in the case of Pearson-networks. Note that the above-described algorithm was adopted from and discussed in detail in Jiang et al. (2010).

*Nonlinearity* was tested by using a surrogate population of signal pairs generated by phase randomization (Theiler et al., 1992). Fourier-transformation of the original pair of signals yielded phase spectra that were shuffled by the same random permutation sequence for all time series, prior to being transformed back into the time domain. This procedure resulted in the destruction of the nonlinear interdependencies between the signals, while the linear dependencies remained preserved (Prichard and Theiler, 1994). The original MI values were compared to the distribution of surrogate MI populations, and the presence of nonlinearity was confirmed if *MI*_*original*_ > μ(*MI*_*surrogate*_) + 2σ(*MI*_*surrogate*_), where μ denotes the mean and σ denotes the standard deviation.

**FIGURE 2:**
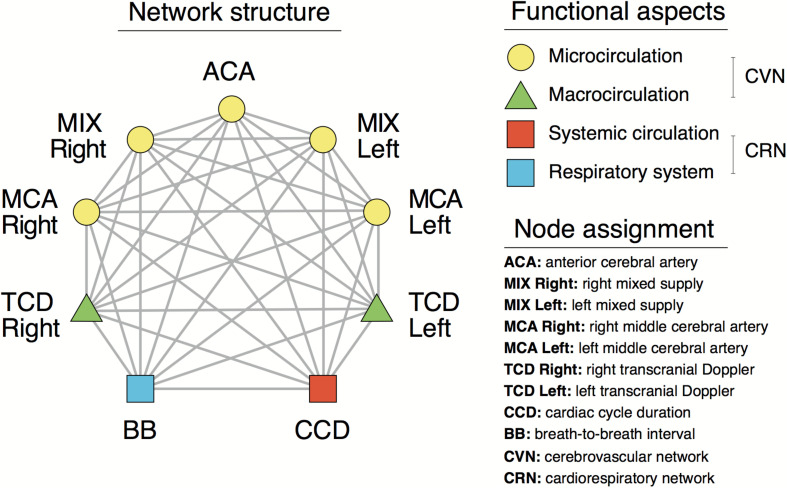
Structural and functional aspects of the cardiorespiratory and cerebrovascular networks.

Based on these relationships, cardiorespiratory-cerebrovascular networks were reconstructed, yielding a Pearson- and an MI-network for both the low- and high-frequency ranges separated at 0.4 Hz. The resampled CCD and BB signals were used as nodes associated with the dynamics in the cardiorespiratory system. Blood flow velocity in the middle cerebral artery (MCA) and the preprocessed NIRS signals were regarded as nodes of the cerebrovascular network. To reduce redundancies within NIRS records we reconstructed networks for 16-channel data first and then combined channels according to the scheme displayed in Figure 1B. This process enables the restructuring of the entire network—from a limited number of nodes—excluding channels with poor signal quality. Thus, the obtained measures of statistical dependencies were then averaged across NIRS-channels corresponding to different vascular territories as follows. The obtained measures of statistical dependencies were then averaged across NIRS-channels corresponding to different vascular territories in the following manner (Figure 1B). Channels in the most lateral position (2–2 on each side) were considered as measuring changes in brain cortex mainly supplied by the middle cerebral arteries (MCAs), the four channels around the midline were considered belonging to supply territories of anterior cerebral arteries (ACAs), while the regions probed by the remaining 4–4 channels were considered receiving perfusion both from MCA and ACA (MIX) on the left and right side, respectively. Ultimately, this arrangement resulted in seven nodes representing macro- (TCD) and microcirculation (NIRS) dynamics in the brain (Figure 2). The interactions within this network were evaluated separately between cardiorespiratory and cerebrovascular networks (CRN) and within the cerebrovascular networks (CVN).

### Statistical Tests

Descriptive statistics are reported as μ ± σ. The continuous variables were analyzed for normality using Shapiro-Wilk test, homogeneity of variances was checked by Levene’s test. If the null hypothesis of normality was rejected, we performed Friedman test to evaluate the effect of change in body position. If the assumption of sphericity was found to be violated (according to Mauchley-test), we used Greenhouse-Geisser correction in order to adjust the signals for lack of sphericity. Repeated measures ANOVA was performed for evaluating the dependence of the relationship parameters on the different states brought about by the experimental maneuvers within the same subject (i.e., resting, stand-up, sit-down). The within-subject factor had 32 levels (30 resting, 1 stand-up, 1 sit-down). Significant differences were identified with Dunnett *post-hoc* test by comparing the obtained *p*-values to a preset level of significance: α_*s*_ = 0.05, if *p* < α_*s*_ also held for the repeated measures ANOVA (using stand-up or sit-down as control conditions). The false discovery rate was controlled by the Benjamini-Hochberg procedure at level α_*s*_ in case of multiple comparisons (Benjamini and Hochberg, 1995). Statistical analyses were carried out in Statistica (TIBCO Software Inc., Palo Alto, Californa, United States) version 13.4.

## Results

### Changes in Physiological Parameters Upon Standing Up and Sitting Down

The average values of arterial blood pressures, cardiac cycle duration and breath-to-breath interval for each period are summarized in Table 1. In the resting state, these values were calculated as averages of the 30 selected segments. Systolic blood pressure (SBP) showed a marked, transient reduction (*p* = 0.0007, confirmed by *post-hoc* tests, too) exerting a major influence on changes of mean arterial blood pressure that followed a similar pattern with non-significant effect of changing body position. Conversely, diastolic blood pressure was slightly higher after standing up, which further increased after sitting down. Cardiac cycle duration changed in the same direction as SBP and MAP via the high-pressure baroreflex mechanism. Accordingly, the heart rate (the inverse of CCD) was elevated in the standing position and was reduced upon sitting down. Breath-to-breath intervals were not affected by postural changes significantly.

**TABLE 1:** Hemodynamic and respiratory parameters of the participating subjects, ^#^ indicates significant difference compared with the resting group.

*n = 9*	Resting	Stand-up	Sit-down
SBP (mm Hg)	124.7 ± 7.7	97.7 ± 10.5^#^	116.3 ± 16.0
DBP (mm Hg)	73.0 ± 10.7	75.7 ± 11.4	81.6 ± 12.4
MAP (mm Hg)	88.9 ± 8.0	84.3 ± 11.3	94.0 ± 14.3
CCD (ms)	824 ± 55	696 ± 94	736 ± 68
BB (s)	3.8 ± 0.8	3.8 ± 0.4	3.8 ± 0.4

**FIGURE 3:**
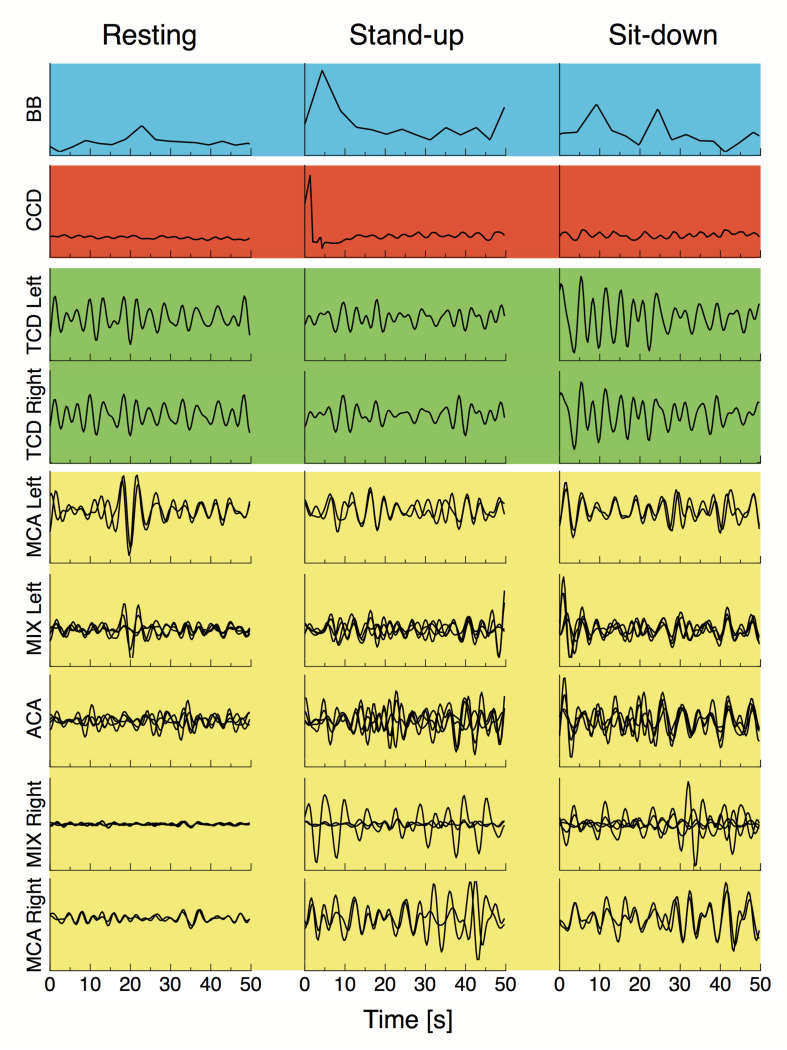
Breath-to-breath interval (BB), cardiac cycle durations, preprocessed blood flow velocity signals acquired from the middle cerebral artery (TCD Left and TCD Right) and preprocessed NIRS signals from different vascular territories are displayed for a representative subject in the resting state, and right after standing up and sitting down. All signals were resampled at 3 Hz and bandpass filtered in the 0.4–1.5 Hz range, blood pressure fluctuations were regressed from all cerebral hemodynamic (i.e., macro- and microcirculatory) signals as described in “Materials and Methods” section. Background colors of the signal panels are the same used in Figure 2.

After standing up, cerebral blood flow velocities showed a decrease in both MCAs, which restored gradually in the sit-down period. Simultaneously, average HbT concentration (both with and without CBSI) lowered in the majority of cortical regions in the time window of observation, but these changes were not significant due to large variability (Table 2). Representative preprocessed physiological time series acquired in steady state, stand-up and sit-down periods are shown in Figure 3.

**TABLE 2:** Cerebrovascular variables derived from NIRS- and TCD-measurements.

	Resting	Stand-up	Sit-down
TCD Left (cm/s)	64.2 ± 1.4	61.0 ± 11.4	65.0 ± 4.3
TCD Right (cm/s)	64.1 ± 1.3	60.9 ± 7.9	64.5 ± 4.5
MCA_Left Δ[HbT] (μM)	1.17 ± 2.69	−0.04 ± 4.78	−0.77 ± 5.65
MIX_Left Δ[HbT] (μM)	0.056 ± 0.072	−0.298 ± 0.390	0.123 ± 1.243
ACA Δ[HbT] (μM)	0.243 ± 0.586	−0.315 ± 1.335	−0.332 ± 1.809
MIX_Right Δ[HbT] (μM)	0.089 ± 0.160	0.161 ± 0.889	−0.033 ± 0.811
MCA_Right Δ[HbT] (μM)	0.119 ± 0.193	−0.030 ± 0.388	−0.265 ± 0.603

### Effect of Postural Changes on Cardiorespiratory-Cerebrovascular Networks

**FIGURE 4:**
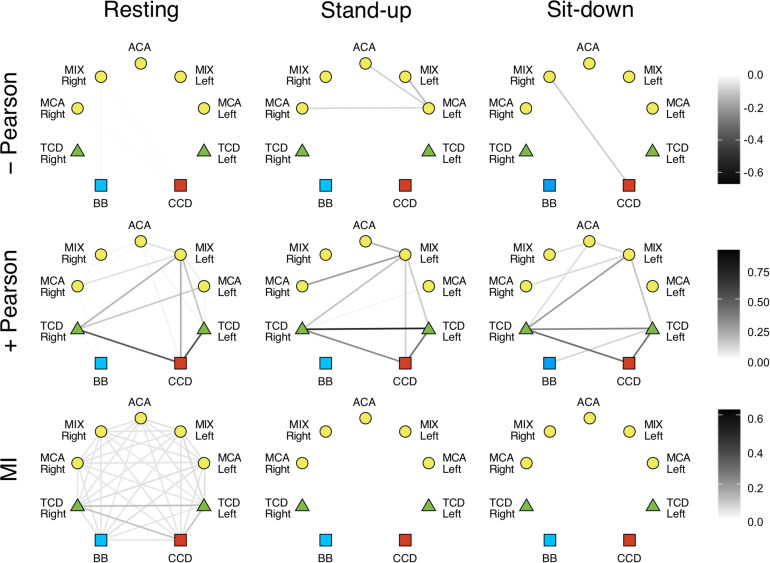
Cardiorespiratory-cerebrovascular networks reconstructed for the low-frequency range (0.02–0.4 Hz) after surrogate thresholding. Connection strength was assessed using Pearson correlation analysis (upper panels: negative correlations, middle panels: positive correlations) and mutual information analysis (MI, lower panels) and is coded in grayscale. CCD, Cardiac cycle duration; BB, Breath-to-breath interval; TCD, transcranial Doppler (denoting the BFV signal recorded from MCA); ACA, anterior cerebral artery; MCA, middle cerebral artery; MIX, NIRS channels sampling regions supplied by ACA and MCA.

**FIGURE 5:**
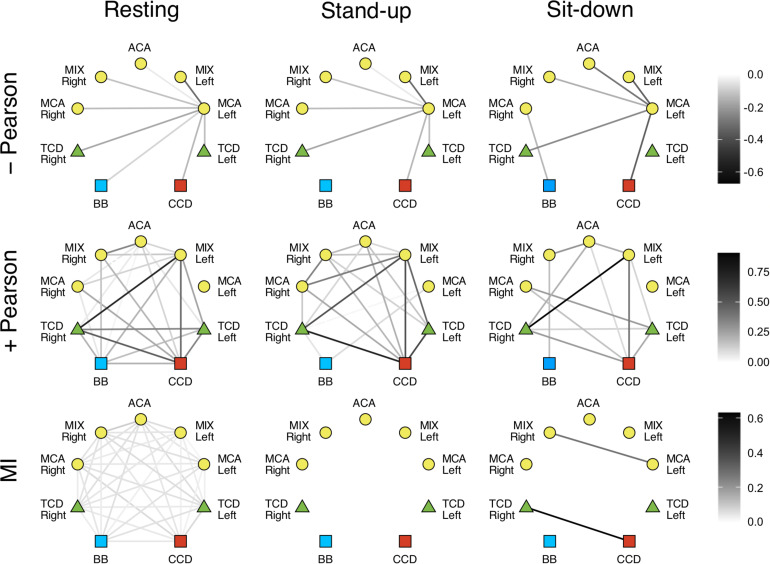
Cardiorespiratory-cerebrovascular networks reconstructed for the high-frequency range (0.4–1.5 Hz) after surrogate thresholding. Strength of connection was assessed using Pearson correlation analysis (upper panels: negative correlations, middle panels: positive correlations) and mutual information analysis (MI, lower panels) and is coded in grayscale. CCD, Cardiac cycle duration; BB, Breath-to-breath interval; TCD, transcranial Doppler (denoting the BFV signal recorded from MCA); ACA, anterior cerebral artery; MCA, middle cerebral artery; MIX, NIRS channels sampling regions supplied by ACA and MCA.

In the LF range, the majority of Pearson coefficients were significant (i.e., it was possible to distinguish them from those obtained from signal pairs following an uncorrelated bivariate normal distribution) in the resting state (∼77%), which decreased after the postural change (stand-up: 73%, sit-down: 59%). In contrast, a much smaller fraction of *r* values were found significant in the HF range (resting: 36%, stand-up: 31%, sit-down: 31%). The majority of significant coefficients indicated positive correlation (*r* > 0). Corresponding networks determined at different measurement conditions did not differ, as shown on the upper panels of Figures 4, 5. Although the strengths of several relationship showed a notable decrease in the low-frequency range (CCD vs. MCA Left, TCD Left vs. MCA Left, TCD Left vs. MIX Right, TCD Right vs. MIX R, MIX L vs. MIX R), after changes in body position (*p* < 0.05) these were not significant taking multiple comparisons into account (false discovery rate correction at level 0.05).

We also investigated the qualitative nature of correlation based on the change of *r*’s sign; for example, if a previously correlated process becomes uncorrelated/anticorrelated or vice versa. Coupled dynamics between CCD and BB in the LF range became uncorrelated after standing up which was not restored until the end of the measurement in both frequency ranges. In the upright posture, a loss of correlation was found between the interaction of respiration with cerebrovascular processes except for TCD Right and MCA Left only in the LF range, where the positive correlations were preserved. The linear interactions were perturbed more by standing up in the HF range followed by a partial restoration in terms of nature of correlation. As to the LF range, we found a rather delayed effect of postural changes: three and nine functional connections in cerebrovascular network were altered—in terms of *r*’s sign—after standing up and sitting down, respectively. It is noteworthy that hemoglobin signal only from the MCA left showed an anticorrelated dynamics mainly in the LF range that was preserved after postural changes.

Phase randomization tests indicated that nonlinear coupling within the investigated processes was only present in the resting state. Nonlinear dependence was found only for a few connections in the LF networks (CCD vs. TCD Right and between ACM Left and MIX Right) after sitting down. However, according to surrogate testing, it was completely absent in the HF networks following postural changes. Due to weak coupling, as indicated by low MI values even in the resting condition, the effect of standing up and sitting down was non-significant for the following relationships: BB vs. TCD (both), BB vs. MCA Right, CCD vs. TCD (both), CCD vs. MIX Left, CCD vs. ACA, TCD Left vs. (TCD Right, MCA Left, MIX Left, MCA Right), TCD Right vs. (MCA Left, MCA Right), MCA Left vs. (MIX Left, ACA, MIX Right), MIX Left vs. ACA, ACA vs. MCA Right, MIX Right vs. MCA Right (the statistical power averaged across connections was 0.642, effect size: 1.010). However, in the HF networks the absence of nonlinearity was seen associated with a significant effect of postural changes when compared with resting condition for all pairs of examined physiological processes (average statistical power was 0.943, effect size: 2.458).

## Discussion

In this study, we investigated the effect of orthostatic stress on the cardiorespiratory-cerebrovascular network. We confirmed that sudden postural changes did not have a significant impact on the cardiorespiratory-cerebrovascular network topology as defined by linear interactions, neither in the low- nor in the high-frequency range; nevertheless, a clear tendency was seen for several connections. In contrast, we demonstrated vanishing nonlinear interactions in the investigated coupled dynamics upon standing up and sitting down clearly distinct from what was observed in the resting state. In spite of preserved homeostatic regulations aimed at maintaining arterial blood pressure and cerebral blood flow, the postural challenge resulted in a complete dissolution of these physiological networks comprising of nonlinear interactions among their components.

Correlated and anticorrelated linear dependencies were more abundant in LF networks, while the majority of connections showed uncorrelated dynamics in the HF range, especially after postural changes. Although none of the connections in the resting Pearson-networks averaged between subjects were statistically different, when compared across different states (Figures 4, 5, upper left panel), these qualitative changes still do provide insight into the response of the CRN and CVN to orthostatic stress. Hence, we propose that the obtained linear relationship parameters in the three states feature the coupled dynamics of the examined processes that were influenced *directly* by standing up/sitting down and *indirectly* by physiological regulations. Importantly, these patterns of changes show a remarkable difference when comparing the statistical interdependencies of breath-to-breath intervals and the rest of the links in the Pearson-networks associated with heart rate and those of cerebral macro- and microcirculation. Accordingly, during orthostatic challenge respiratory dynamics became swiftly independent of the cardiac and cerebrovascular dynamics. In contrast, heart rate maintained its relationship with the rest of the examined physiological processes except in the HF range, where the beat-to-beat regulation of blood pressure is a prevailing mechanism. Thus, postural changes affected the cerebrovascular network differently, suggesting the role of frequency-specific response in the pressure autoregulation of cerebral blood flow (Giller and Mueller, 2003). In line with that, further investigation of LF range revealed that postural changes had marginally more impact on the 0.15–0.4 Hz then oscillations of 0.06–0.15 or 0.02–0.06 Hz (spectral ranges commonly used in analysis of NIRS records). However, since the lower frequencies are less represented in 50 s records, the observed differences between the physiological states rather refer to the HF and LF range applied in this study. Given that blood pressure fluctuations were eliminated from cerebrovascular signals consisting our physiological networks, the observed changes reflect an indirect effect of ABP changes and ABP-independent effects of orthostatic stress on BFV and HbT dynamics assessed by TCD and NIRS, respectively. The origin of the two-tiered responses of the Pearson-network can also be attributed in part to this preprocessing step (see differences of surrogate testing results) as well as to the impact of frequencies inherent to various mechanisms mediating systemic effects of postural changes, which are more prevalent in the LF range (Orini et al., 2012).

The cerebrovascular dynamics observed during orthostatic challenge emerges from a combination of promptly developing passive and active changes manifesting with a delay, particularly evoked by arterial baroreflex mechanism. Accordingly, we observed a noticeable reduction of SBP accompanied by reflex tachycardia (Table 1), which among our healthy young participants in the initial phase of the stand-up period should be regarded as part of the underlying physiological adaptation to the orthostatic challenge (Stewart, 2013). The extent of this drop is comparable to the value of ∼30 mmHg reported in a similar study (Olufsen et al., 2005) and should be distinguished from that of orthostatic hypotension as defined by consensus statement (Freeman et al., 2011; Moloney et al., 2020). Since standing up resulted in a larger decrease of blood flow velocity in the MCA than that of ABP, the cerebrovascular resistance (estimated as ABP/BFV with cross-section area of MCA assumed remaining unaltered during the maneuver; Aaslid et al., 1989) must have led to perturbed cerebral hemodynamics invoking pressure autoregulation of global cerebral blood flow. The observed changes in our study (Table 2) correspond well with previous findings of Sorond et al. (2009), who also examined cardio- and cerebrovascular adaptation to orthostatic stress among elderly normo- and hypertensive subjects.

Analysis of transfer function between blood pressure and blood flow velocity changes (Panerai et al., 1999) showed that standing up yielded gain values dropping clearly below 1 (consistent with the case of negative feed-back regulation), especially for slow oscillations of these signals. This indicates the presence of pressure autoregulation that is more effective in the low-frequency range, similarly to what Sammons et al. (2007) have found with the same method. Moreover, the moderate coherence between MAP and BFV in both frequency ranges implies the presence of nonlinearity or the influence of a hidden linear regressor, which could explain their even weaker relationship below 0.4 Hz due to vasomotion. As to NIRS measurements, tissue HbT concentrations decreased after standing up in parallel with a reduction of blood pressure, which is an apparent short-term passive effect of postural change. In case of measurements carried out in the resting and sit-down positions, we found that HbT changes (which without CBSI follow changes in cerebral blood volume) were rather anti- or uncorrelated with blood pressure changes. Since cerebral autoregulation dilates brain vessels in case of decreased blood pressure, cerebral blood volume—at unchanged tissue hematocrit—increases marked by an elevated tissue HbT levels. Thus the latter should be regarded as a signature of cerebrovascular reactivity—that is assessed in a 5 min time window (Lee et al., 2009)—although in the evolving phase of this compensation.

Given that MI is a model-free measure (Steuer et al., 2002), we also reconstructed mutual information networks to evaluate the contribution of nonlinear dynamics. MI analysis depicts a consistent effect of orthostatic stress in both frequency ranges either on the group or the individual level. Lower panels of Figures 4, 5 show that standing up disintegrates the MI networks, which practically remains unchanged until the end of the measurement. In fact, phase randomization emphasized the qualitative differences between different physiological states yielding a remarkable contrast while it was not evident for the significance of Pearson correlations. Thus, to a large extent, it is the surrogate testing approach applied to these networks that accounts for this pattern. Recall that MI captures both linear and nonlinear dependencies (Smith, 2015) and that we performed a variant of phase randomization that tests nonlinearity, only (Prichard and Theiler, 1994). In other words, in spite of a preserved linear dependency also captured by MI, the absence of nonlinear coupled dynamics rendered the weight of this functional link in the corresponding physiological network to 0. Taken together, our analytical framework demonstrated vanishing nonlinear interactions in response to orthostatic challenge, which prompts questions for future research about the nature of the physiological mechanisms at play.

The duration of the cardiac and respiratory cycle dynamics are interrelated via several intricate relationships (Voss et al., 2009). One of the most apparent patterns in the coupled dynamics of these oscillatory systems is known as respiratory sinus arrhythmia, which refers to a decrease in CCD during inspiration and increase in CCD during expiration due to altered parasympathetic tone. This periodic influence was present in the observed dynamics throughout the measurement independent of postural changes, most likely due to multiple uncontrolled factors apart from those associated with the vagal activity (e.g., change in tidal volume). Moreover, Bartsch and Ivanov (2014) demonstrated a phase synchronization between heart rate and respiratory rate, which is a nonlinear form of interaction contributing to the fine structure of the examined physiological networks. Thus, the absence of coupled nonlinear dynamics confirmed by our surrogate testing (phase randomization) also excludes phase synchronization between CCD and BB after standing up and sitting down, which results in impaired coordination between these physiological systems playing a role in homeostatic mechanisms. Nonlinear interactions between cardiovascular signals have been shown to be suppressed by the baroreflex mechanism after head-up tilt resulting in their simplification and increased predictability (Faes and Nollo, 2006). Numerous studies evidenced the presence of nonlinearities in the cerebral hemodynamics (Panerai et al., 1999; Mitsis et al., 2004). Mitsis et al. (2002) identified frequency-dependence of dynamic cerebral autoregulation and its attenuation during orthostatic stress (Mitsis et al., 2006). Our findings fundamentally agree with these observations since the measured physiological processes became more independent after postural changes indicating their weakening regulations.

Evaluating both linear and nonlinear interactions is indispensable for a detailed reconstruction of physiological networks (Faes et al., 2015). We characterized cardiorespiratory-cerebrovascular networks by combining qualitative assessment of linear and non-linear dependencies with simple (Pearson-correlation) or model-free measures (mutual information) of coupling, which does not directly allow for the assessment of causality. There is an abundance of such bivariate methods that have been utilized in recent studies of physiological networks (Bashan et al., 2012; Bartsch et al., 2015; Zanetti et al., 2019), for a review see Schulz et al. (2013). The relatively short time series (150 data points) were insufficient for adopting alternative bivariate measures such as symbolic transfer entropy (Dickten and Lehnertz, 2014; Lucchini et al., 2020), Granger causality (Faes et al., 2008) or measures of scale-free coupled dynamics (Stylianou et al., 2021) with suitable surrogate testing capable of identifying causal relationships (Schreiber and Schmitz, 1996). Adequate data representation is also necessary for using fractal models based on capturing spatio-temporal cross-dependencies between coupled physiological processes in order to identify physiological networks by utilizing fractional differencing operators (Xue et al., 2016; Bogdan, 2019). Hence, we preferred using stochastic measures known to be insensitive to short data and thus offering a more flexible description of physiological networks compared to deterministic models under our experimental conditions. Our framework captured fundamental changes in the topology of the CRN-CVN brought about by orthostatic stress and in future studies it is of high interest to investigate its directional couplings. Ultimately, it can be readily applied to any kind of physiological networks either for exploration or identifying effects of perturbation where a more elaborate model cannot be utilized due to unmet assumptions about representation or statistical properties of data.

As to limitations of our study, it is important to note that because the maneuvers were inherently associated with perturbations generating large, transient motion artifacts in the physiological records, we had to exclude the very early phase of the postural challenge from the analysis. While the subsequently recorded data (right after postural changes) became artifact-free, we could secure a sufficiently long segment for the network reconstruction, thus our analysis necessarily skipped the time window associated with the immediate dynamic autoregulatory response combating the very early effects of the perturbations. Furthermore, the short time spent in the perturbated states is of another concern. With a longer stand-up period, one might have been able to observe whether the MI cardiorespiratory-cerebrovascular network recovered to its resting-state topology at all. Although the final number of participants was relatively low it was still comparable to that in other human network physiological studies (Faes et al., 2015) in addition to being balanced by the within-subject design of our experimental protocol. Finally, given that electrophysiological data (such as electroencephalography) was not collected during our measurements, this circumstance did not allow us to disentangle subsystems within the investigated physiological network with respect to brain activity changes.

Globally adequate delivery of nutrients and oxygen matching the needs of brain tissue is a vital homeostatic mechanism established by a fine-tuned interaction between respiration and systemic regulations of circulation. Hence, despite the above limitations, incorporating components of central, macro- and microcirculation into a physiological network is a novel adaptation of the network physiology concept, which could contribute to a deeper understanding of healthy regulatory mechanism maintaining homeostasis. These dynamics show intricate dependencies that were found challenged by orthostatic stress, which raises questions about linear and nonlinear network topologies associated with physiological perturbations in other organ systems, too.

## Conclusion

In the present study, we found that postural changes induced radical topological reorganization in the nonlinear cardiorespiratory-cerebrovascular network. The interdependencies between cardiac, respiratory and cerebrovascular dynamics showed a two-tiered response: non-significant changes in the Pearson and a marked weakening in the mutual information network topologies reconstructed from linear and nonlinear coupled dynamics, respectively. The disruption of nonlinear networks suggests that the complexity of key homeostatic mechanisms maintaining cerebral hemodynamics and oxygenation is indeed susceptible to physiological perturbations such as orthostatic stress.

## Data Availability Statement

The datasets generated for this study – entitled “Two-tiered response of cardiorespiratory-cerebrovascular networks to orthostatic challenge” – can be requested from the corresponding author or can be found in the PhysioNet online repository under the same name: http://physionet.org/content/.

## Ethics Statement

The studies involving human participants were reviewed and approved by the Regional and Institutional Committee of Science and Research Ethics, Semmelweis University. The patients/participants provided their written informed consent to participate in this study.

## Author Contributions

PM performed the measurements and analysis and wrote the first draft of the study. ZN carried out the measurements. FR performed the preprocessing of the data. IP helped with the data collections. AH and FR wrote the scripts for time series analysis. OS created the figures and contributed to the statistical assessments. RD helped with the experimental setup. DB provided the research instruments and guidance in the data collection. AE conceptualized and organized the study, and contributed to the manuscript revisions and data visualizations. All authors reviewed and edited the manuscript, and approved its final version.

## Conflict of Interest

The authors declare that the research was conducted in the absence of any commercial or financial relationships that could be construed as a potential conflict of interest.
