# Corrigendum: Two-Tiered Response of Cardiorespiratory-Cerebrovascular Network to Orthostatic Challenge

**DOI:** 10.3389/fphys.2021.685417

**Published:** 2021-04-21

**Authors:** Peter Mukli, Zoltan Nagy, Frigyes Samuel Racz, Istvan Portoro, Andras Hartmann, Orestis Stylianou, Robert Debreczeni, Daniel Bereczki, Andras Eke

**Affiliations:** ^1^Department of Physiology, Semmelweis University, Budapest, Hungary; ^2^Vascular Cognitive Impairment and Neurodegeneration Program, Oklahoma Center for Geroscience and Healthy Brain Aging, Department of Biochemistry and Molecular Biology, University of Oklahoma Health Sciences Center, Oklahoma City, OK, United States; ^3^Institute of Translational Medicine, Semmelweis University, Budapest, Hungary; ^4^Institute for Globally Distributed Open Research and Education (IGDORE), Stockholm, Sweden; ^5^Department of Neurology, Semmelweis University, Budapest, Hungary; ^6^Department of Radiology and Biomedical Imaging, Yale University School of Medicine, New Haven, CT, United States

**Keywords:** network physiology, orthostatic stress, cardiorespiratory, cerebrovascular, near-infrared spectroscopy, transcranial Doppler, nonlinear, surrogate testing

In the published article, there was an error in affiliations 1 and 2. For affiliation 1, instead of “Vascular Cognitive Impairment and Neurodegeneration Program, Oklahoma Center for Geroscience and Healthy Brain Aging, Department of Biochemistry and Molecular Biology, University of Oklahoma Health Sciences Center, Oklahoma City, OK, United States,” it should be “Department of Physiology, Semmelweis University, Budapest, Hungary.” For affiliation 2, instead of “Department of Biochemistry and Molecular Biology, Oklahoma Center for Geroscience and Healthy Brain Aging, University of Oklahoma Health Sciences Center, Oklahoma City, OK, United States,” it should be “Vascular Cognitive Impairment and Neurodegeneration Program, Oklahoma Center for Geroscience and Healthy Brain Aging, Department of Biochemistry and Molecular Biology, University of Oklahoma Health Sciences Center, Oklahoma City, OK, United States.”

The authors apologize for this error and state that this does not change the scientific conclusions of the article in any way. The original article has been updated.

